# Test your knowledge and understanding

**Published:** 2017-03-03

**This page is designed to help you to test your own understanding of the concepts covered in this issue, and to reflect on what you have learnt.**

**Figure:**
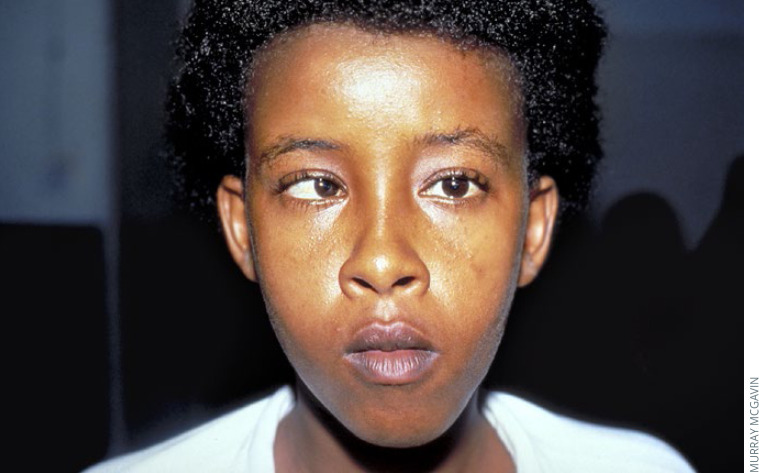
Is this a sudden onset of a 6th nerve palsy or a longstanding esotropia? This boy may have a longstanding right esotropia (if he is looking straight) or a left 6th nerve palsy (if he is attempting to look left).

We hope that you will also discuss the questions with your colleagues and other members of the eye care team, perhaps in a journal club. To complete the activities online – and get instant feedback – please visit **www.cehjournal.org**

**Tick ALL that are TRUE**

**Question 1 Anisocoria:**
  □ **a**. May be due to an oculomotor (3rd nerve) palsy
  □ **b**. May be due to bilateral optic atrophy
  □ **c**. May occur in association with partial ptosis
  □ **d**. May be due to mydriatics
  □ **e**. May be associated with heterochromia of the iris
**Question 2 Homonymous hemianopia:**
  □ **a**. Means loss of field of vision in one eye with normal visual field in the other eye
  □ **b**. May be due to a stroke (CVA) affecting the occipital cortex
  □ **c**. Can cause difficulty in reading, eating and driving
  □ **d**. May be due to an orbital lesion causing proptosis
  □ **e**. May occur during a migraine attack
**Question 3 Diplopia:**
  □ **a**. May follow a head injury with reduced abduction on eye movements
  □ **b**. Can be due to myasthenia gravis
  □ **c**. If it persists when one eye is closed, then it is due to a lesion in the brain
  □ **d**. May be associated with proptosis
  □ **e**. The separation of images is greatest in the direction of action of the paralysed muscle

## ANSWERS

1. a, c, d and e. Anisocoria is a difference in size between the two pupils. Third nerve palsy may paralyse the constrictor muscle of the iris and cause a dilated pupil on the affected side and can also cause ptosis. Horner's syndrome is due to paralysis of the sympathetic fibres which serve the pupil dilator muscle and may cause a constricted pupil on the affected side together with partial ptosis and sometimes depigmentation of the iris (heterochromia). In optic atrophy (unilateral or bilateral) the pupils are the same size, although they will have a poor reflex reaction to light.
2. b, c and e. Homonymous hemianopia means half the field of vision is lost in both eyes – the nasal field in one eye and the temporal field in the other. The result is loss of half the bilateral field of vision. It may occur with any lesion affecting the optic tract, radiation or cortex.
  As half the bilateral visual field is lost, only half the page, half the plate and half the driving field of vision is seen. Occasionally it can occur with a migraine attack, usually recovering but not always.
3. a, b, d and e are correct. A head injury may cause a palsy of the 6th cranial nerve which supplies the lateral rectus. Myasthenia gravis causes weakness of muscles on use and can lead to diplopia and ptosis. Monocular diplopia is due to ocular conditions e.g. media opacities. Proptosis, particularly associated with dysthyroid eye disease can cause diplopia. The diplopia is greatest in the direction of action of the paralysed muscle.

**Reflective learning**

Please visit **www.cehjournal.org** to complete the online ‘Time to reflect’ section.

